# Targeted Disruption of E6/p53 Binding Exerts Broad Activity and Synergism with Paclitaxel and Topotecan against HPV-Transformed Cancer Cells

**DOI:** 10.3390/cancers14010193

**Published:** 2021-12-31

**Authors:** Marta Celegato, Lorenzo Messa, Chiara Bertagnin, Beatrice Mercorelli, Arianna Loregian

**Affiliations:** 1 Department of Molecular Medicine, University of Padua, 35121 Padua, Italy; marta.celegato@unipd.it (M.C.); lorenzo.messa@unipd.it (L.M.); chiara.bertagnin@unipd.it (C.B.); beatrice.mercorelli@unipd.it (B.M.); 2 Clinical Microbiology and Virology Unit, Padua University Hospital, 35121 Padua, Italy

**Keywords:** cervical cancer, head-and-neck cancer, HPV, targeted therapy, synergy, Paclitaxel, Topotecan

## Abstract

**Simple Summary:**

The identification of new specific anti-human papillomavirus (HPV) drugs is highly needed, as HPV-induced cancers still represent a significant medical issue. The aim of this study was to analyze in more detail the therapeutic potential of a compound, Cpd12, that acts by blocking the binding between HPV E6 oncoprotein and cellular tumor suppressor p53. We demonstrated that by blocking such an interaction, driven by highly conserved residues among oncogenic HPVs, Cpd12 exhibits broad activity against cervical cancer cell lines infected by different HPV genotypes and HPV-positive head-and-neck cancer cells. Interestingly, Cpd12 also showed the ability to inhibit cancer cell migration and to increase the activity of chemotherapeutic drugs such as taxanes and topoisomerase inhibitors. These findings improve the knowledge about the in vitro efficacy of Cpd12, paving the way to preclinical studies to develop new therapeutic strategies against HPV-induced tumors.

**Abstract:**

High-risk human papillomaviruses (HR-HPV) are the etiological agents of almost all cervical cancer cases and a high percentage of head-and-neck malignancies. Although HPV vaccination can reduce cancer incidence, its coverage significantly differs among countries, and, therefore, in the next decades HPV-related tumors will not likely be eradicated worldwide. Thus, the need of specific treatments persists, since no anti-HPV drug is yet available. We recently discovered a small molecule (Cpd12) able to inhibit the E6-mediated degradation of p53 through the disruption of E6/p53 binding in HPV16- and HPV18-positive cervical cancer cells. By employing several biochemical and cellular assays, here we show that Cpd12 is also active against cervical cancer cells transformed by other HR-HPV strains, such as HPV68 and HPV45, and against a HPV16-transformed head-and-neck cancer cell line, suggesting the possibility to employ Cpd12 as a targeted drug against a broad range of HPV-induced cancers. In these cancer cell lines, the antitumoral mechanism of action of Cpd12 involves p53-dependent cell cycle arrest, a senescent response, and inhibition of cancer cell migration. Finally, we show that Cpd12 can strongly synergize with taxanes and topoisomerase inhibitors, encouraging the evaluation of Cpd12 in preclinical studies for the targeted treatment of HPV-related carcinomas.

## 1. Introduction

Human papillomavirus (HPV) infections are recognized as one of the major causes of pathogen-related cancer [1]. High-risk (HR)-HPV types, a sub-group of mucosal HPVs, are not only responsible for virtually all cervical cancer cases but also related to other anogenital tumors and a growing percentage of head-and-neck cancers [2,3]. The International Agency for Research on Cancer (IARC) defined at least 12 HPV types as carcinogenic to humans, namely HPV16, 18, 31, 33, 35, 39, 45, 51, 52, 56, 58, and 59 [4]. Among them, HPV16 and HPV18 stand out for their highest carcinogenic capacity [5]. Additionally, HPV-66, 68, and 73 are now considered as probable carcinogens and are therefore included in the group of high-risk strains [6]. To date, three different prophylactic vaccines exist, namely Cervarix (covering against HPV16 and 18), quadrivalent Gardasil (covering against HPV16, 18, and two low-risk strains), and nonavalent Gardasil (covering against HPV16, 18, 31, 33, 45, 52, 58, and two low-risk strains). Although prophylactic vaccination campaigns significantly reduced the incidence of cervical cancer [7], the vaccination coverage significantly differs among countries [8]. In addition, recent studies indicated that after a quadrivalent-based vaccination campaign, the prevalence of non-vaccine high-risk genotypes did not significantly decline [9,10]. Therefore, in the next decades the ongoing prevention strategies employed worldwide will not likely eradicate HPV-related cancers. Moreover, treatments are highly needed, particularly for the multitude of already infected individuals who are at risk of developing tumors, but, unfortunately, no specific anti-HPV drugs exist yet to be used as targeted anticancer drugs in the clinics. Currently, standard-of-care treatments are based on surgery, radiotherapy, and chemotherapy with platinum-based agents and other drugs [11]. However, these approaches are invasive and cytodestructive, and are often associated with toxicity problems [12]. Hence, the need for specific anti-HPV drugs for therapeutic intervention remains of paramount importance.

The mechanisms by which HR-HPV infections lead to malignant cell transformation rely on the activities of two viral oncoproteins, E6 and E7, which cooperate to transform the cell during a persistent infection [13]. Targeting E6 is an appealing therapeutic approach for the treatment of HPV-induced cancers because its suppression can reactivate different tumor-suppressive pathways. The targeted disruption of the E6/E6AP binding, E6/Caspase 8 interaction, and E6/p53 association through small-molecules have been the most pursued and attractive strategies for drug development, as they can lead to the rescue of cellular targets committed to degradation, reactivation of the extrinsic apoptosis, and the rescue of p53-mediated signaling, respectively [11]. All of these approaches have the potential to be therapeutically effective. On this line, we recently discovered a small molecule—Cpd12—able to inhibit the E6-mediated degradation of p53 through the disruption of the direct interaction of E6 with p53. Cpd12 was shown to give rise to a robust p53-mediated transcriptional program that resulted in an antitumoral activity against HPV16- and 18-positive cervical cancer cells [14]. However, given the abundance of different high-risk genotypes, assessing whether a targeted drug-like molecule has the potential to be effective against multiple genotypic variants is critical. In this study, we wished to investigate whether Cpd12 maintains the same antitumoral activity against cancer cells harboring HR-HPV strains other than HPV16 and 18 and against HPV-transformed cells of non-cervical origin. We show that Cpd12 is similarly active against HPV68-positive ME180 and HPV45-positive MS751 cervical cancer cells, as well as against the HPV16-positive SCC152 head-and-neck cancer cell line. Moreover, our data indicate that the combination of Cpd12 with standard chemotherapeutic drugs potentiates the antitumor activity of the latter by exerting a synergistic effect, representing a promising future therapeutic strategy for the treatment of HPV-related cancers.

## 2. Materials and Methods

### 2.1. Compounds

Test compounds **3** (Cpd3) and **12** (Cpd12) were purchased from SPECS (compound ID AN-465/43461703 and AJ-292/42490180, respectively). RITA was purchased from Cayman Chemical. Cisplatin, Paclitaxel, and 5-Fluorouracil were purchased from Selleckem. Topotecan was purchased from MedChem Express. All compounds, except for Cisplatin, were dissolved in DMSO and stock solutions were stored at −20 °C. Cisplatin was dissolved in water and stock solution was stored at 4 °C.

### 2.2. Cell Lines

HPV-positive HeLa (HPV18), CaSki (HPV16), SiHa (HPV16), ME180 (HPV68), MS751 (HPV45), and HPV-negative C33A cervical cancer cells, HPV-positive SCC152 (HPV16) and HPV-negative FaDu head-and-neck cancer cells, and human foreskin fibroblasts (HFF) were all from the American Type Culture Collection (ATCC, Manassas, VA, USA). Cell lines, except for SCC152 cells, were maintained in Dulbecco modified Eagle’s medium (DMEM; Thermo Fisher Scientific, Waltham, MA, USA) supplemented with 10% fetal bovine serum (FBS; Thermo Fisher Scientific, Waltham, MA, USA), 100 U/mL penicillin, and 100 mg/mL streptomycin sulfate (P/S; Thermo Fisher Scientific, Waltham, MA, USA). SCC152 cells were cultured in minimum essential medium Eagle (EMEM; ATCC, Manassas, VA, USA), supplemented with 10% fetal bovine serum (FBS; Thermo Fisher Scientific, Waltham, MA, USA), 2 mM L-Glutamine (Gibco, Thermo Fisher Scientific, Waltham, MA, USA), MEM non-essential amino acids solution (NEAA; Gibco, Thermo Fisher Scientific, Waltham, MA, USA), and 50 ug/mL Gentamicin (Gibco, Thermo Fisher Scientific, Waltham, MA, USA). Cells were kept in culture at 37 °C in a humidified atmosphere with 5% CO_2_ for no more than 4 weeks (approximately 8–10 passages) and were regularly tested for mycoplasma contamination. 

### 2.3. Cell Viability Assays

To test compounds’ cytotoxicity, 1 × 10^4^ ME180, 1.5 × 10^4^ MS751, 4 × 10^4^ C33A, 1.5 × 10^4^ SCC152, 1 × 10^4^ FaDu, and 1 × 10^4^ HFF cells were seeded into 96-well plates and the next day treated with increasing concentrations (1.95–250 µM) of each compound in duplicate. Cell viability was then determined at 48 h post-treatment by the 3-(4,5-dimethylthiazol-2-yl)-2,5-diphenyl tetrazolium bromide (MTT) method as previously reported [14]. 

### 2.4. Two-Dimensional Clonogenic Assays

ME180, MS751, C33A, SCC152, and FaDu cells were seeded in 6-well plates at a density of 250, 1000, 150, 750, and 500 cells/well, respectively, and treated with Cpd3 (50 µM) or Cpd12 (25–50 µM) for 72 h. Next, cell medium was replaced with fresh medium containing the same compounds concentrations and cells were incubated for a further 72 h. Successively, cells were cultured for one additional week in fresh medium without compounds. Finally, cell colonies were fixed and stained (0.05% crystal violet, 1% PFA, 1% methanol in PBS). Aggregates with > 50 cells were scored as colonies and counted.

### 2.5. Tumor Spheroid Formation Assays

To evaluate the effect of Cpd12 on spheroid formation, MS751 (1 × 10^3^ cells/well) and SCC152 (1 × 10^3^ cells/well) cells were seeded on 24-well Nunclon Sphera plates (Thermo Fisher Scientific, Waltham, MA, USA) in the presence of DMSO, Cpd3, or Cpd12 (50 µM). Cells were cultured in Keratinocyte-SFM (Gibco, Thermo Fisher Scientific, Waltham, MA, USA) supplemented with 10 ng/mL b-FGF (Gibco, Thermo Fisher Scientific, Waltham, MA, USA), 10 ng/mL EGF (Gibco, Thermo Fisher Scientific, Waltham, MA, USA), and B27 (Gibco, Thermo Fisher Scientific, Waltham, MA, USA). The medium was changed every day in the presence of fresh compounds and tumor sphere growth was monitored daily for 7 days.

### 2.6. Western Blot Analysis

ME180, MS751, and SCC152 cells seeded at 2 × 10^5^ per well in 6-well plates were treated with test compounds for 48 h. Western blotting was performed as previously described [15] and cell lysates were analyzed with anti-p53 (DO-1, 1:4000; Santa Cruz Biotechnology, Dallas, TX, USA) and anti-β-actin (A5441, 1:8000; Sigma-Aldrich, St. Louis, MO, USA) mouse primary antibodies. Immunocomplexes were detected with goat anti-mouse antibodies conjugated to horseradish peroxidase (sc-2055, 1:2000; Santa Cruz Biotechnology, Dallas, TX, USA). Uncropped Western blots can be found in the Supplementary Materials. 

### 2.7. Gene Expression Analysis by Quantitative Real-Time PCR (qPCR)

To analyze the effects of test compounds on the transcription of a panel of p53 target genes, ME180, MS751, and SCC152 cells were seeded at 2 × 10^5^ in 6-well plates and treated for 24 h. Total RNA was then purified with RNA Purification Plus kit (Norgen Biotek, Thorold, ON, Canada) and cDNA was generated from RNA (1.5 µg) using random primers (Applied Biosystems, Waltham, MA, USA) and M-MLV reverse transcriptase (Applied Biosystems). The qPCR process was performed with SYBR green (Applied Biosystems, Waltham, MA, USA) on a 7900 HT Fast Real-Time PCR System (Applied Biosystems, Waltham, MA, USA). A complete list of primer sequences was previously reported [14].

### 2.8. Cytofluorimetric and Immunofluorescence Analysis for Cell-Cycle Phase Determination

ME180, MS751, and SCC152 cells (2 × 10^5^) were seeded in 6-well plates and the next day were treated with Cpd3 or Cpd12 (50 μM). After 24 h, cell cycle progression was evaluated by propidium iodide (PI) staining and flow cytometric analysis on a FACSCalibur Flow Cytometer (BD Biosciences, Franklin Lakes, NJ, USA). The percentage of cells in each phase of the cell cycle was calculated using the Mod-Fit LT software.

Immunofluorescence studies were performed as previously described [16], with minor modifications. Briefly, 3 × 10^4^ HeLa, CaSki, SiHa, ME180, MS751, and SCC152 cells seeded on glass 12-mm-diameter coverslips in 24-well plates were treated with DMSO or Cpd12 (50 μM) for 24 h and then fixed in 4% PFA solution (Santa Cruz Biotechnology, Dallas, TX, USA). Ki67 was immunostained using mouse anti-Ki67 (MAB4190, 1:500, Millipore, Merck KGaA, Darmstadt, Germany) primary antibody, except for CaSki cells, wherein Ki67 was visualized using mouse anti-Ki67 (sc-23900, 1:500, Santa Cruz Biotechnology, Dallas, TX, USA) primary antibody. Nuclear staining was performed by incubating cells with DRAQ5™ (BioStatus Ltd., Shepshed, UK) 1:2000 in PBS for 20 min at room temperature. Images were acquired with a Nikon A1RSi laser scanning inverted confocal microscope equipped with NIS-Elements Advanced Research software (Nikon Instruments Inc., Tokyo, Japan) using a 60× ocular objective. For all samples, at least five random fields of view were acquired performing Z-stack acquisitions with an average of 20 planes per image. Ki67 and DRAQ5 fluorescence intensities were quantified from maximum intensity projections using ImageJ 2.0. Cell-cycle phases were determined as a function of Ki67 and DRAQ5 fluorescence intensities as described by Miller and colleagues [17].

### 2.9. In Situ Staining of Senescence-Associated β-Galactosidase Activity, Autofluorescence Analysis, and Detection of Apoptosis

To detect senescence-associated β-galactosidase activity, ME180, MS751, and SCC152 cells (2.5 × 10^3^) were seeded into 24-well plates and the next day cells were treated for 72 h with test compounds. Cells were then cultured for a further 8 days in fresh medium. Finally, cells were fixed with 4% PFA in PBS, washed with PBS and incubated at 37 °C for 36 h with SA-β-galactosidase-staining solution (1 mg/mL 5-bromo-4-chloro-3-indolyl β-D-galactopyranoside [X-gal], 5 mM K_3_Fe[CN]_6_, 5 mM K_4_Fe[CN]_6_, 2 mM MgCl_2_ in PBS pH 6). Blue-green cell cytoplasmic staining, indicative of cell senescence due to an increase of lysosomal β-galactosidase activity, was examined under phase-contrast microscopy, and representative images were taken.

Cellular autofluorescence was determined by incubating ME180, MS751, and SCC152 cells (5 × 10^4^) for 120 h on 6-well plates in the presence of test compounds (50 μM). Cells were then harvested and changes in autofluorescence were assessed by flow cytometry detection in the FITC (green) channel using a LSR II flow cytometer (BD Biosciences, Franklin Lakes, NJ, USA). The acquired data were analyzed by the Flowing software.

To evaluate apoptosis induction, ME180, MS751, and SCC152 cells were incubated on 6-well plates for 48, 72, 96, or 120 h (seeding 2 × 10^5^, 1 × 10^5^, 7.5 × 10^4^, or 5 × 10^4^, respectively) in the presence of each test compound. Cells were then harvested and AnnexinV-FITC binding along with PI staining (eBioscience™ Annexin V Apoptosis Detection Kit FITC, Thermo Fisher Scientific, Waltham, MA, USA) was analyzed by flow cytometry using a LSR II flow cytometer (BD Biosciences, Franklin Lakes, NJ, USA). Data analysis was carried out using the FACSDiva software (BD Biosciences, Franklin Lakes, NJ, USA).

### 2.10. Cell Migration Assays

Cell migration was assessed by the scratch wound healing assay and by transwell assay. For wound healing assay, HeLa, CaSki, and ME180 cells (4 × 10^5^, 5 × 10^5^, and 3 × 10^5^, respectively) were seeded in 6-well plates. When the cells were confluent, two wound lines were made by scratching the cellular monolayer with a 10-μL pipette tip. The culture medium and detached cells were then removed. The scratched wells were washed three times with PBS and then cells were treated for 24 h with test compounds in fresh medium with 0.5% FBS to avoid cell proliferation and restrict the filling of the scratch to migrated cells. The wound closure was recorded before treatment and after 12, 24, and 48 h by taking representative images under phase-contrast microscopy.

For transwell assays, HeLa cells were treated with test compounds for 24 h. Next, 1 × 10^5^ cells were resuspended in 100 µL of culture medium without serum and then reseeded into the upper chamber of a transwell insert (8 µm pore size; Corning Inc., Corning, NY, USA) arranged into a well of 24-well plate containing 600 µL of medium with 10% FBS. After incubating at 37 °C for 24 h, cells on the top surface of the insert were removed by gentle wiping with a cotton swab, and the cells that migrated to the bottom surface of the insert were fixed and stained in 4% PFA solution containing 0.1% crystal violet for 30 min. Representative images of migrated cells were taken under phase-contrast microscopy.

### 2.11. Drug Combination Studies

To evaluate the combined effects of Cpd12 with different chemotherapeutic drugs on HPV-positive cancer cells, we first determined the IC_50_ values for Cisplatin, Paclitaxel, and Topotecan in HeLa, CaSki, and MS751 cells, and for Cisplatin, Paclitaxel, and 5-Fluorouracil in SCC152 cells by MTT assays. Cells were then treated for 48 h with each drug alone or in combination at equipotent ratios, using 0.125-, 0.25-, 0.5-, 1-, and 2-fold their respective IC_50_ values. Cytotoxicity was evaluated by the MTT assay. The 2-drug combination effects were assessed by calculating the combination index (CI) using the Chou-Talalay method [18] according to mass-action law based dynamic theory computed in the CalcuSyn software version 2.0 (Biosoft, Cambridge, UK).

### 2.12. Statistical Analysis

Data were analyzed using GraphPad Prism 8 (GraphPad Software Inc., San Diego, CA, USA).

## 3. Results

### 3.1. Cpd12 Selectively Affects the Viability and Proliferation of HPV-Transformed Cells of Both Cervical and Head-and-Neck Origin and Rescues p53 Levels and Transcriptional Activity

We have recently identified a drug-like compound (named hereafter Cpd12, Figure S1) endowed with antitumoral activity selective for HPV-transformed cancer cells as it binds and inhibits the viral oncoprotein E6 to rescue p53 protein levels [14]. We previously determined the activity of this compound against cancer cells transformed by HPV16 and HPV18 genotypes, the two most prevalent viral strains in cervical cancer specimens [19]. With the goal to study the activity of this small molecule against other E6 genotypic variants, we took advantage of commercially available cervical cancer cell lines bearing HPV strains other than HPV16 and HPV18. However, the commercial availability of such cell lines is solely restricted to ME180 and MS751 cells bearing HPV68 and HPV45, respectively. We also included in the analysis an HPV-transformed cell line of non-cervical origin, i.e., SCC152 head-and-neck cancer cells (HPV16-positive) derived from the hypopharynx [20], in order to characterize the activity of this compound against HPV-positive cancer cells independently of the tissue of origin. 

**Figure 1:**
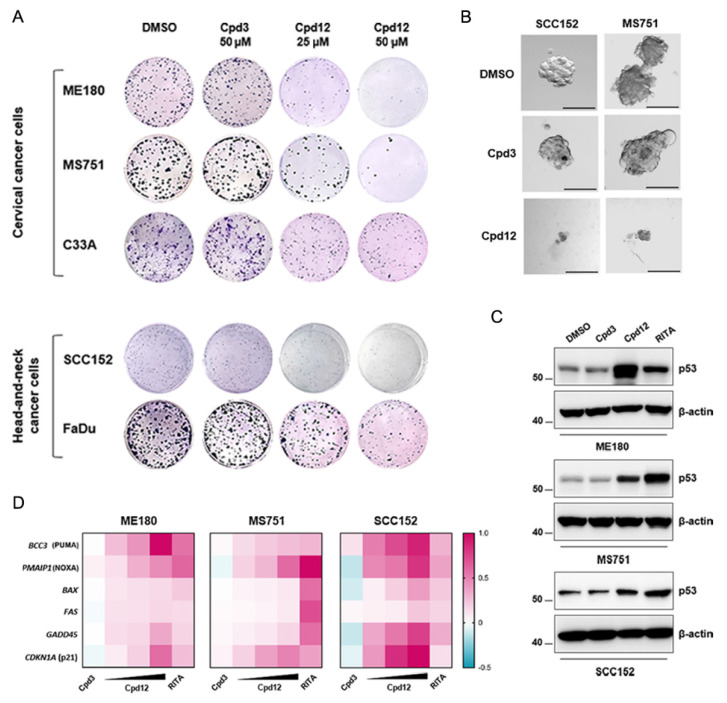
Cpd12 selectively affects the viability and proliferation of HPV-transformed cells of both cervical and head-and-neck origin and rescues p53 levels and transcriptional activity. (**A**) 2D colony formation assays were performed to evaluate the effects of Cpd12 on the proliferation of ME180, MS751, C33A, SCC152, and FaDu cells. Images are representative of one out of three independent experiments. (**B**) The effect of Cpd12 (50 µM) on the formation of 3D tumor spheroids was evaluated by culturing MS751 and SCC152 cells as tumor spheroids from single-cell suspensions. Representative images were taken at 7 days post-treatment using a bright-field inverted microscope (100× magnification). Scale bar 50 µm. (**C**) The effects of test compounds on the E6-mediated degradation of p53 were assessed by Western blotting in ME180 (HPV68) and MS751 (HPV45) cervical cancer cells, and in SCC152 (HPV16) head-and-neck cancer cells treated for 48 h. RITA (5 µM for ME180 and SCC152 cells, and 1 µM for MS751 cells) was used as a positive control. (**D**) The effects of Cpd12 on the transcription of a panel of p53 target genes *BBC3*, *PMAIP1*, *BAX*, *FAS*, *CDKN1A*, and *GADD45* were assessed by qPCR in ME180, MS751, and SCC152 cells treated with Cpd12 (10, 25, and 50 µM) for 24 h. The heatmaps show the normalized mean fold-change values of compound-treated ME180, MS751, and SCC152 cells compared to their relative DMSO-treated control cells. In all panels, cells treated with the inactive small molecule Cpd3 or DMSO were included as negative controls.

Initially, we wished to validate that Cpd12 was specifically active against these HPV-transformed cells. Therefore, we initially performed MTT and colony formation assays to measure both the viability and the proliferation of ME180, MS751, and SCC152 cells exposed to Cpd12 treatment. Two HPV-negative cancer cell lines of both cervical (C33A cells) and head-and-neck (FaDu cells) origin were included for comparison. In addition, primary fibroblasts (HFF) were also included as a non-transformed negative control in MTT assays. Remarkably, Cpd12 significantly affected both the viability and proliferation of HPV-positive ME180, MS751, and SCC152 cells with minor effects on HPV-negative counterparts (Figure 1A and Figure S2A–C). In MTT assays, the IC_50_ values of ME180, MS751, and SCC152 cells were 12.4 ± 0.3 µM, 12.4 ± 0.9 µM, and 7.5 ± 0.5 µM, respectively (Figure S2A,B), which are very similar to those previously estimated for HeLa, CaSki, and SiHa cells [14]. Conversely, C33A, FaDu, and HFF cells were significantly less susceptible to Cpd12 treatment, with at least 6-fold higher IC_50_ values (93 ± 14 µM, 72 ± 8.6 µM. and >250 µM, respectively; Figure S2A,B). In parallel, the proliferation of ME180, MS751, and SCC152 cells was markedly reduced, with minor effects against C33A and FaDu cancer cells (Figure 1A and Figure S2C), strengthening our previous evidence that Cpd12 acts preferentially and selectively against HPV-transformed cells. The anticlonogenic activity of Cpd12 in standard 2D proliferation assays was paralleled to compound activity also in 3D spheroid formation assays. In fact, Cpd12 blocked the generation of MS751- and SCC152-derived tumor spheroids from single-cell suspensions, demonstrating its activity also against the cancer-stem cell population of these two cell lines (Figure 1B), as we previously reported for HeLa cells [15]. Unfortunately, ME180 did not generate cervospheres under these experimental conditions (data not shown) and could not be used for this analysis. Following these initial results, Western blot analysis confirmed that Cpd12 induced the upregulation of endogenous p53 protein levels in ME180, MS751, and SCC152 cells, albeit to different extents (Figure 1C and Figure S3A). In line with the observed rescue of p53, treatment of these three cell lines with Cpd12 induced a dose-dependent transcriptional upregulation of some bona fide p53-target genes, particularly *BBC3*, *PMAIP1*, *CDKN1A,* and *GADD45*, with differential inductions according to the cell line examined (Figure 1D and Figure S3B). The inactive small molecule Cpd3, emerged from the initial screening searching for anti-E6/p53 compounds and used as a negative control [14], did not show any activity during the course of these experiments as expected (Figure 1A–D, Figure S2B,C and Figure S3A,B). Conversely, the small molecule RITA, which was shown to block p53 degradation in cervical cancer cells likely through DNA damage [21,22], was used as a positive control of p53 rescue (Figure 1C,D).

### 3.2. The Cell-Cycle Arrest Induced by Cpd12 Occurs in the G1 Phase

**Figure 2:**
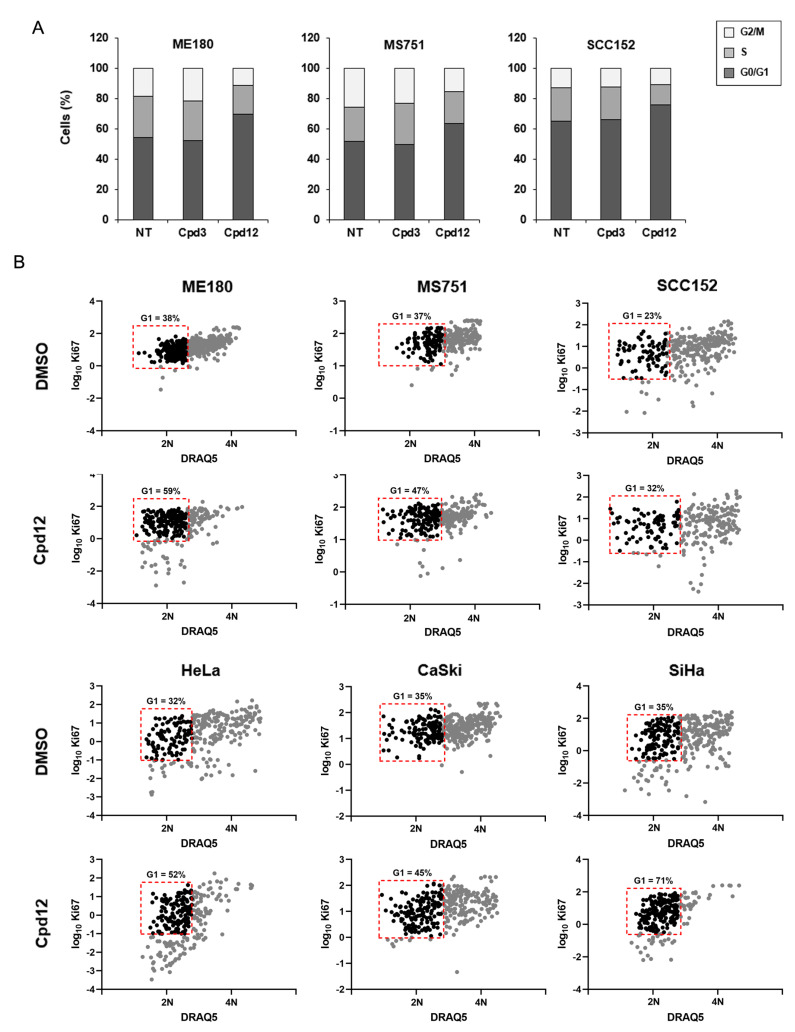
The cell-cycle arrest induced by Cpd12 occurs in the G1 phase. (**A**) The effects of Cpd12, the inactive small molecule Cpd3, or DMSO, on cell cycle modulation were evaluated by cytofluorimetric analysis of HPV-positive cervical (ME180 and MS751) and head-and-neck (SCC152) cancer cells treated for 24 h and then stained with PI. Bar graphs represent the percentage of cells in the different phases of the cell cycle (G0/G1, M, G2/S) as the mean of three independent experiments. (**B**) Cell-cycle distributions of HPV-positive cervical (ME180, MS751, HeLa, CaSki, and SiHa) and head-and-neck (SCC152) cancer cells treated with Cpd12 (50 µM) or DMSO for 24 h were determined through immunofluorescence assays. Cells in either the G0, G1, S, G2, or M phase were determined as a function of Ki67 and nuclear staining, and fluorescence intensities were quantified from maximum intensity projections of Z-stack images acquired with a 600× magnification. Total numbers of cells were: n_DMSO_ = 463 and n_Cpd12_ = 304 for ME180 cells, n_DMSO_ = 303 and n_Cpd12_ = 316 for MS751 cells, n_DMSO_ = 314 and n_Cpd12_ = 267 for SCC152 cells, n_DMSO_ = 303 and n_Cpd12_ = 270 for HeLa cells, n_DMSO_ = 388 and n_Cpd12_ = 330 for CaSki cells, and n_DMSO_ = 336 and n_Cpd12_ = 244 for SiHa cells. Nuclei were stained with DRAQ5. 2N: diploid DNA content, 4N: tetraploid DNA content.

We have previously shown that the p53-mediated response triggered by Cpd12 treatment induces a block in the G0/G1 phases of the cell cycle of HeLa cells, leading mainly to premature senescence [14]. By analyzing cellular DNA content through propidium iodide staining, cytofluorimetric analysis confirmed that treatment with Cpd12 increases the amount of ME180, MS751, and SCC152 cells in the G0/G1 phases, whereas, as expected, Cpd3 has no effect (Figure 2A). To further dissect the mechanism of action of Cpd12 beyond this phenomenon, we wished to investigate whether the compound acts by either blocking proliferating cells in the G1 phase or by increasing the fraction of quiescent cells in G0 through stimulation of their exit from the cell cycle. To this aim, we employed an imaging-based single-cell analysis to indirectly assess the phases of the cell cycle according to the intensity of the proliferation marker Ki67 that was recently reported to appear in G1 and then increase progressively through S and M phases [17]. We compared Ki67 fluorescence intensities with the relative DNA content of each cell determined through DRAQ5 staining to profile the sub-populations of ME180, MS751, and SCC152 cells in the G0, G1, S, G2, and M phases. We included in the analysis also HeLa, CaSki, and SiHa cells as their response to Cpd12 treatment was not previously analyzed with this imaging-based experimental platform [14]. First, we validated the approach in HeLa cells and confirmed that Ki67 immunofluorescence coupled with nuclear staining can be used to distinguish the phases of the cell cycle (Figure S4A,B), as reported [17]. We have previously shown that Cpd12 mainly promotes premature senescence in HeLa cells [14], and it has been reported that with the emergence of senescence, which is classically considered to occur after at least some days of exposure to stress [23], Ki67 disappears independently of the cell-cycle phase in which cells were blocked [24,25]. Thus, we analyzed cells treated with Cpd12 or DMSO at 24 h post-treatment, a time point before Ki67 staining could start disappearing in cells exposed to Cpd12 treatment. Intriguingly, when comparing the distributions of Cpd12-treated ME180, MS751, SCC152, HeLa, CaSki, and SiHa cells with DMSO-treated controls, we observed that in all samples treatment with Cpd12 significantly increased the number of cells in G1, rather than G0 (Figure 2B). Therefore, our results collectively indicate that Cpd12, by inhibiting the E6-mediated degradation of p53, blocks proliferating HPV-transformed cells in G1 to dictate a downstream p53-dependent response. 

### 3.3. Cpd12 Treatment Induces Senescent and Apoptotic Responses in Both Cervical and Head-and-Neck Cancer Cells

**Figure 3:**
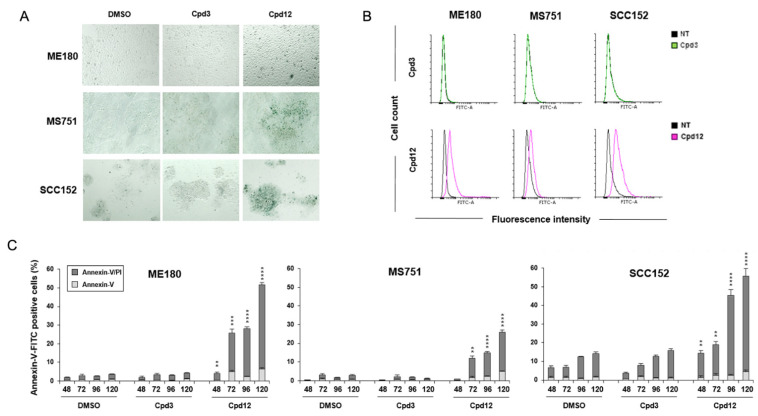
Cpd12 treatment induces senescent and apoptotic responses in both cervical and head-and-neck cancer cells. (**A**) SA-β-gal staining of ME180, MS751, and SCC152 cells treated with Cpd12. Representative images taken using a bright-field inverted microscope (100× magnification) are shown. (**B**) Autofluorescence ofME180, MS751, and SCC152 cancer cells was assessed by flow cytometry after treatment with Cpd12 as described in Methods. Histograms are representative of one out of three independent experiments. (**C**) Apoptosis induction in ME180, MS751, and SCC152 cells treated with Cpd12 for 48, 72, 96, and 120 h. Values in the bar graphs represent the mean ± SD of at least three independent experiments. Data were analyzed by a two-way ANOVA followed by Tukey’s multiple comparison test; **, *p* < 0.01; ***, *p* < 0.001; ****, *p* < 0.0001, Cpd12 versus DMSO. In all panels, cells treated with the inactive small molecule Cpd3 or DMSO were included as negative controls.

We have shown that Cpd12-induced rescue of p53 led mainly to the induction of senescence in HeLa cells, coupled also with an apoptotic response when cells were exposed to a prolonged treatment [14]. Therefore, we sought to confirm that also other HPV-transformed cell lines (ME180, MS751, and SCC152) show similar responses and undergo premature senescence and/or apoptosis in response to Cpd12 treatment. We evaluated the occurrence of senescence through both senescence-associated β-galactosidase (SA-β-gal) staining and cytofluorimetric analysis of the increase of cellular autofluorescence due to the accumulation of age-related pigment lipofuscin [26]. When we exposed ME180, MS751, and SCC152 cells to a 3-day treatment with Cpd12, we observed clusters of cells positive for SA-β-gal staining, a hallmark of senescence (Figure 3A). Accordingly, the autofluorescence of ME180, MS751, and SCC152 cells significantly increased following a 5-day exposure to Cpd12 (Figure 3B). In parallel, we could also measure a significant increase in the fraction of apoptotic cells for all three cell lines (Figure 3C). The effect was already markedly evident upon a 3-day treatment with Cpd12, with an accumulation of apoptotic cells that ranged from approximately 20% for MS751 cells to almost 45% and 50% for SCC152 and ME180 cells, respectively, after a 5-day exposure (Figure 3C). These results agree with our previous characterization of the antitumoral effects of Cpd12 in HeLa cells [14] and show that Cpd12 can induce a mixed p53-dependent senescent and apoptotic response also in ME180, MS751, and SCC152 cells.

### 3.4. The Cpd12-Induced Rescue of p53 Blocks the Migration of HPV-Transformed Cells

**Figure 4:**
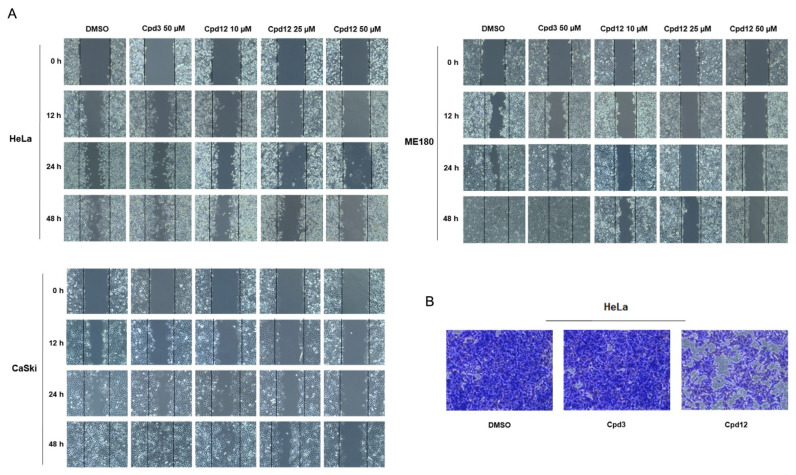
The Cpd12-induced rescue of p53 affects the migration capacity of HPV-transformed cells. (**A**) Confluent monolayers of HeLa, CaSki, and ME180 cervical cancer cells were scraped up two times and then treated with increasing concentration of Cpd12 (10, 25, and 50 µM) in low serum medium. The wound closure was monitored before treatment and after 12, 24, and 48 h, and representative images were taken using a bright-field inverted microscope (100× magnification). (**B**) HeLa cells were treated with Cpd12 (50 μM) for 24 h. Then, cell migration was evaluated by transwell assays after an incubation of other 24 h in Cpd12-free medium. Representative images of migrated cells fixed and colored with crystal violet were taken using a bright-field inverted microscope (100× magnification). In both panels, cells treated with DMSO or Cpd3 (50 μM) were included as negative controls.

Both cervical and head-and-neck HPV-related cancers are known to frequently metastasize, particularly in the liver, lungs, and lymph nodes [27]. Inactivation or loss of p53 functions have been also linked to the biology of cancer cell migration and activated p53 can inhibit the function of different Rho GTPases, limiting the migration capacity of cancer cells and preventing metastasis [28]. Therefore, we expected that Cpd12, by rescuing p53 functions, could also affect the migration potential of HPV-transformed cells. We thus performed wound healing assays to assess the migration rate of HeLa, CaSki, and ME180 cells, which among available HPV-transformed cells are the three cell lines that better spread in culture. Because Cpd12 triggers a downstream p53-mediated senescent and apoptotic response following at least a 3 day treatment (Figure 3A–C), we performed the assays by treating cells for no longer than 24 h to precede the induction of senescence/apoptosis that might interfere with the migration potential. Importantly, when comparing the rate of migration of Cpd12-treated cells with DMSO-treated controls, we indeed observed that HeLa, CaSki, and ME180 cells significantly lost migration capacity when exposed to Cpd12, whereas this effect was not observed upon treatment with Cpd3 (Figure 4A and Figure S5). The effect of Cpd12 on cancer cell migration was also tested in transwell migration assays in which we monitored the spreading of HeLa cells, which is one of the HPV-transformed cell lines that shows a sustained expression of epithelial-to-mesenchymal-related genes that confers high motility and invasive potential [29]. In line with the results obtained with wound healing assays, Cpd12 significantly reduced the migration rate of HeLa cells also in this experimental setting (Figure 4B).

### 3.5. Cpd12 Enhances the Cytotoxic Effect of Clinically-Approved Anticancer Drugs

Standard-of-care treatments for HPV-driven cancers currently rely on the use of radiotherapy and chemotherapy, either alone or in combination. Platinum-based agents and taxanes are among the most frequently chemotherapeutics employed in the clinic to treat HPV-related cancers of both cervical and head-and-neck origin [12]. In addition, other chemotherapeutic drugs are clinically employed, such as Topotecan in the case of cervical cancer [30] and 5-Fluorouracil in the case of locally-advanced head-and-neck tumors [31], usually in combination with platinum-based agents and taxanes. Therefore, we wished to investigate whether Cpd12 could synergize with clinically approved anticancer drugs used to treat HPV-related cancers, in particular Cisplatin, Paclitaxel, Topotecan, and 5-Fluorouracil. To this aim, we performed MTT assays with three representative cervical cancer cell lines (HeLa, CaSki, and MS751 cells) and head-and-neck SCC152 cancer cells to assess the viability of cells exposed to different drug combinations. We initially determined the IC_50_ values of each drug alone (data not shown), in order to combine equipotent doses of Cpd12 with each chemotherapeutic. Importantly, when combined at equipotent molar ratios, we observed an overall good synergism of Cpd12 with all the chemotherapeutic drugs tested (Table 1). In particular, Cpd12 showed strong synergism with Paclitaxel in all cell lines at all tested combinations (Table 1), indicating that the anti-E6 activity of Cpd12 strongly enhances the toxic effect of a microtubule-stabilizer drug. We also observed good synergism with Topotecan in cervical cancer cells, while the combination of Cpd12 with Cisplatin and 5-Fluorouracil showed only a moderate synergism at subtoxic doses (Table 1).

## 4. Discussion

Cancers induced by persistent HPV infection depend on sustained E6/E7 activities for progression and maintenance. This feature intrinsically addresses the development of targeted drugs towards E6 and/or E7 oncoproteins. However, after more than 20 years of preclinical studies, HPV-related cancers are still orphan of targeted drugs. Through a structure-based screening, we have recently discovered a small-molecule compound—Cpd12—that binds within a shallow pocket in the *N*-terminal domain of E6 where key contacts with p53 take place [14]. In our previous study, we conducted the screening using E6 of HPV16 and extended the characterization of the antitumoral properties of Cpd12 to HeLa cells that endogenously express HPV18 E6, thus evaluating Cpd12 activity against the two most prevalent HPV genotypes [19]. However, the indication that several other genotypes are still persisting in the vaccinated population [9,10,32] highlights the strong need of targeted anti-HR-HPV drugs active also against non-vaccine strains. We therefore wished to evaluate the antitumoral activity of Cpd12 in cancer cells transformed by other HR-HPV strains. As several amino acids of the surface-exposed region of E6 targeted by Cpd12 are highly conserved among mucosal HR-HPV genotypes [16], such a high homology suggests that Cpd12 should efficiently target different high-risk E6 variants. Unfortunately, the sole availability of ME180 and MS751 cervical cancer cells in the biotech market limited this study to HPV68 and HPV45, respectively, and future work is required to evaluate Cpd12 activity against other prevalent high-risk strains, such as HPV31 or HPV33. Nevertheless, here we demonstrate that Cpd12 is active against ME180 and MS751 cells endogenously expressing HPV68 E6 and HPV45 E6, respectively. Of note, whereas the nonavalent formulation of Gardasil covers against HPV45 infections, HPV68 is not included in any of the available prophylactic vaccines. In addition, we show that Cpd12 is similarly active against the HPV16-positive SCC152 head-and-neck cancer cell line, indicating that Cpd12 can exert antitumoral activity against HPV-transformed cells of non-cervical origin.

An intriguing result that emerged during this study is the observation that Cpd12 arrests proliferating HPV-transformed cells specifically in the G1 phase (Figure 2B). Collectively, our data thus indicate that Cpd12, by inhibiting the E6/p53 interaction in HPV-transformed cells, leads to p53 rescue and activation to trigger a subsequent block in G1. This is in agreement with the notion that p53 exerts a critical role in the G1/S checkpoint to regulate S phase entry [33]. Although the E6-mediated degradation of p53 should ideally occur at any phase of the cell cycle, our data suggest that targeted disruption of the E6/p53 interaction has major significance during the G1 phase of the cell cycle of HPV-positive cancer cells. Coupled to the block in the G1 phase, we also present evidence that Cpd12 can significantly impact not only on the proliferative potential of HPV-transformed cells, but also on their migration capacity (Figure 4A,B), indicating that targeted reactivation of p53 through E6 inhibition can orchestrate a cascade of antitumoral responses. In a wider perspective, the targeted reactivation of p53 through Cpd12 treatment might have a therapeutic potential also against productive HPV infections in premalignant lesions by exerting a pure antiviral effect. This is supported by previous evidence about the importance of p53 degradation for HPV genome amplification in non-transformed keratinocytes [34], and the fact that keratinocytes harboring HPV genomes with p53-binding-defective E6 mutants were reported to undergo senescence [35]. In this regard, Cpd12 might also be potentially employed as an antiviral drug, although further optimization is likely necessary to increase drug potency.

Downstream of the p53-mediated arrest in the G1 phase, we show that Cpd12 can induce both a senescent and apoptotic response in ME180, MS751, and SCC152 cells, similarly to what we previously observed in HeLa cells upon Cpd12 treatment [14]. The mixed senescent and apoptotic responses measured in ME180, MS751, and SCC152 cells might be explained by the differential upregulation of p53-target genes. Indeed, we observed a significant apoptotic response mainly in Cpd12-treated ME180 cells (Figure 3C), congruent with a marked increased transcription of the pro-apoptotic gene *BBC3* (Figure 1D), whereas MS751 and SCC152 cells underwent mainly premature senescence according to the evident appearance of SA-β-gal-positive cell clusters (Figure 3A).

Finally, this study also indicates that Cpd12 might be effectively used in combination with standard chemotherapeutics in the clinic to treat HPV-related tumors to potentiate the antitumoral activities of the latter (Table 1). Indeed, cumulative knowledge on cancer biology suggests that drug combinations are more likely to cope with tumor complexity compared to single agents. Combination therapy allows for reduction in drug doses, leading to less pronounced side effects and decreasing the emergence of drug resistance [36]. In particular, our in vitro analysis indicates that Cpd12 strongly synergizes with Paclitaxel (Table 1), which acts by stabilizing microtubules and inducing a mitotic catastrophe in dividing cells in a p53-independent manner [37]. In addition, Paclitaxel is known to block cells at the G2/M checkpoint [38]. Therefore, the strong synergism observed might be due to a complementary effect of the drugs, wherein Paclitaxel blocks dividing cells in G2, but Cpd12 blocks the remaining susceptible population in G1. Similarly, Cpd12 shows good synergism with Topotecan, a topoisomerase inhibitor considered to act mainly during the S phase [39] and to delay the G2/M transition and inducing p53-independent cell death [40,41]. Therefore, the Topotecan-induced arrest in either the S or G2 phase could complement the Cpd12-induced arrest in G1, thus explaining the synergism observed (Table 1). According to our data, the combination of Cpd12 with Paclitaxel and Topotecan might allow for the lowering of each individual drug dosage, thus reducing their adverse effects, improve the magnitude of therapy efficacy, and decrease emergence of tumor resistance to the treatment. In contrast, the lower synergism observed with Cisplatin and 5-Fluorouracil can be justified by the mechanisms of action of these drugs. Indeed, both drugs induce DNA damage leading to p53 activation and p53-dependent cell death [42]. Therefore, Cpd12 likely exerts a mild synergism by converging on the same intracellular effector of Cisplatin and 5-Fluorouracil, i.e., p53. 

## 5. Conclusions

In conclusion, although a limitation of this study is that no in vivo xenograft experiments have been performed yet to investigate whether Cpd12 can reduce tumor growth, our data warrant future preclinical studies involving the combination of Cpd12 with taxanes and topoisomerase inhibitors for the treatment of HPV-related cancers.

## Figures and Tables

**Table 1:** Analysis of the effect of the drug combination on HPV-positive cervical and head-and-neck cancer cells viability.

		Combination Index (CI) ^a^		
	Drug Combination at Equipotent Ratio (Fold of IC_50_^b^)	Cisplatin + Cpd12	Paclitaxel + Cpd12	Topotecan + Cpd12
**HeLa**	0.125×	0.457 ± 0.091	0.196 ± 0.095	0.494 ± 0.084
	0.25×	0.797 ± 0.103	0.329 ± 0.104	0.520 ± 0.169
	0.5×	0.677 ± 0.116	0.355 ± 0.085	0.442 ± 0.102
	1×	0.901 ± 0.117	0.482 ± 0.107	0.663 ± 0.134
**CaSki**	0.125×	0.895 ± 0.271	0.104 ± 0.060	0.348 ± 0.057
	0.25×	0.705 ± 0.087	0.180 ± 0.079	0.398 ± 0.102
	0.5×	0.577 ± 0.125	0.268 ± 0.108	0.386 ± 0.021
	1×	0.914 ± 0.162	0.516 ± 0.187	0.624 ± 0.026
**MS751**	0.125×	0.832 ± 0.092	0.161 ± 0.072	0.649 ± 0.221
	0.25×	0.930 ± 0.035	0.197 ± 0.037	0.785 ± 0.071
	0.5×	0.814 ± 0.219	0.274 ± 0.052	0.472 ± 0.165
	1×	0.653 ± 0.103	0.279 ± 0.132	0.372 ± 0.136
		**Cisplatin + Cpd12**	**Paclitaxel + Cpd12**	**5-Fluorouracil + Cpd12**
**SCC152**	0.125×	0.484 ± 0.124	0.135 ± 0.021	0.636 ± 0.074
	0.25×	0.598 ± 0.097	0.242 ± 0.035	0.635 ± 0.081
	0.5×	0.796 ± 0.235	0.438 ± 0.026	0.643 ± 0.037
	1×	0.691 ± 0.179	0.355 ± 0.051	0.431 ± 0.062

^a^ Combination Index (CI), obtained by computational analysis with Calcusyn software. Reported values represent means ± SD of data derived from *n* ≥ 3 independent experiments in duplicate. Drug combination effect defined as: strong synergism for 0.1 < CI < 0.3; synergism for 0.3 < CI < 0.7; moderate synergism for 0.7 < CI < 0.85; slight synergism for 0.85 < CI < 0.9; nearly additive for 0.9 < CI < 1.1, according to Chou et al. [18]. ^b^ Fold decrease of the IC_50_ for drug combinations yielding an equipotent ratio between the doses of the two combined drugs. The CI values were determined by MTT assays treating the different cell lines with each drug alone (for reference) or in combination. Both drugs were combined at their respective IC_50_ values (1×) or at equivalent fractions (0.5×, 0.25×, 0.125×).

## Data Availability

This study did not generate datasets.

## Supplementary Materials

The following are available online at https://www.mdpi.com/article/10.3390/cancers14010193/s1, Figure S1: Predicted binding mode of Cpd12 to E6 protein upon Molecular Dynamics simulation, Figure S2: Cpd12 selectively affects the viability and proliferation of HPV-transformed cells of both cervical and head-and-neck origin, Figure S3: Cpd12 rescues p53 levels and transcriptional activity in both cervical and head-and-neck cancer cells, Figure S4: The cell-cycle arrest induced by Cpd12 occurs in the G1 phase, Figure S5: The Cpd12-induced rescue of p53 affects the migration capacity of HPV-transformed cells. Uncropped Western blots can be found in the Supplementary Materials.
