# Supplementary material for: Targeted Disruption of E6/p53 Binding Exerts Broad Activity and Synergism with Paclitaxel and Topotecan against HPV-Transformed Cancer Cells
Source: Cancers (Basel). 2021 Dec 31;14(1):193. doi: 10.3390/cancers14010193 (PMC8750593; doi:10.3390/cancers14010193)
Supplement: Supplementary file 1 [file cancers-14-00193-s001.zip › cancers-1494294-supplementary materials.pdf]

Supplementary Materials

Targeted disruption of E6/p53 binding exerts broad activity and synergism with Paclitaxel and Topotecan against HPV-transformed cancer cells.

Marta Celegato, Lorenzo Messa, Chiara Bertagnin, Beatrice Mercorelli, and Arianna Loregian

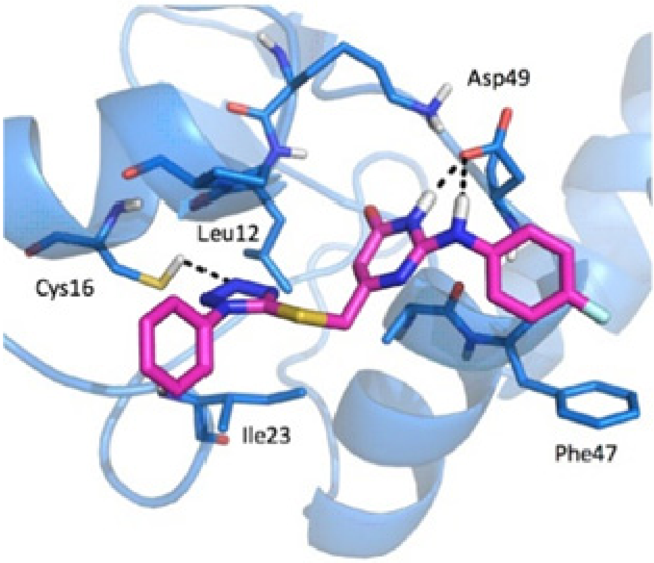

Figure S1. Predicted binding mode of Cpd12 to E6 protein upon Molecular Dynamics simulation.

Cpd12, the residues of E6 protein covering the pocket, and the waters mediating the protein-ligand interaction are shown in capped sticks; the E6 protein is represented in cartoons. Hydrogen bonds are depicted as dashed lines. The E6 residues interacting with the ligand are indicated. Reproduced from ref. [14] with permission from Elsevier, Copyright 2020.

A

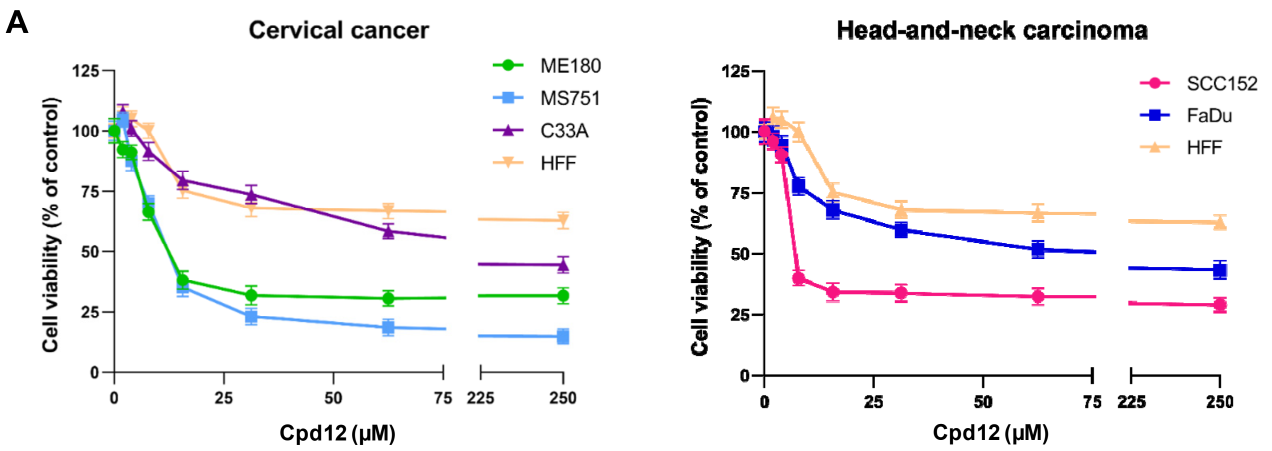

B

	IC50 (µM)	
	Cpd12	Cpd3
Cervical cancer		
HeLa* (HPV18)	11.8 ± 2.6	>250
CaSki* (HPV16)	16.3 ± 4.8	>250
SiHa* (HPV16)	26.7 ± 6.9	>250
ME180 (HPV68)	12.4 ± 0.3	>250
MS751 (HPV45)	12.4 ± 0.9	>250
C33A (HPV -)	93 ± 14	>250
Head-and-neck cancer		
SCC152 (HPV16)	7.5 ± 0.5	>250
FaDu (HPV -)	72 ± 8.6	>250
HFF	>250	>250

C

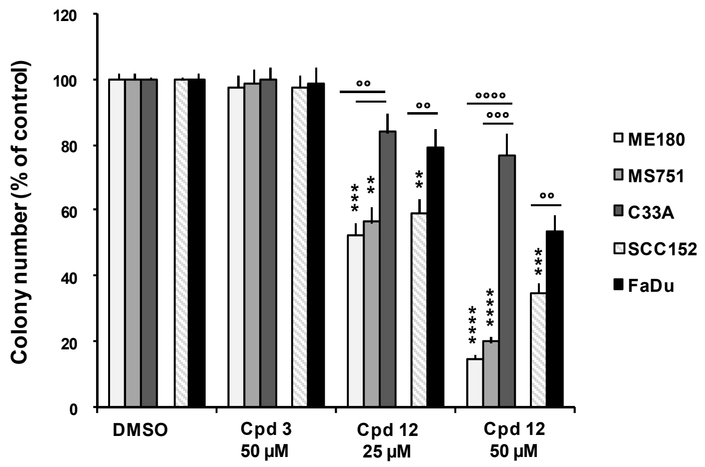

Figure S2. Cpd12 selectively affects the viability and proliferation of HPV-transformed cells of both cervical and head-and-neck origin.

(A) The effects of Cpd12 on the viability of HPV-positive (ME180, MS751) and HPV-negative (C33A) cervical cancer cells, HPV-positive (SCC152) and HPV-negative (FaDu) head-and-neck cancer cells, and non-transformed fibroblasts (HFF) were assessed by MTT assays after 48 h of treatment.

(B) IC_{50} values (50% Inhibitory Concentration, the compound concentration that inhibits 50% cell viability) represent the mean ± SD of $n \geq 3$ independent experiments performed in duplicate. *, the indicated IC_{50} values for HeLa, CaSki, and SiHa cells are those reported in Celegato et al. [14].

(C) Bar graph representing the mean percentage of colony number ± SD of three independent experiments relative to the images shown in Figure 1A. Data were analyzed by a two-way ANOVA

followed by Tukey's multiple comparison test. **, $p < 0.01$; ***, $p < 0.001$; ****, $p < 0.0001$ Cpd12 *versus* DMSO; °°, $p < 0.01$; °°°, $p < 0.001$; °°°°, $p < 0.0001$ C33A or FaDu cells *versus* HPV-positive cells.

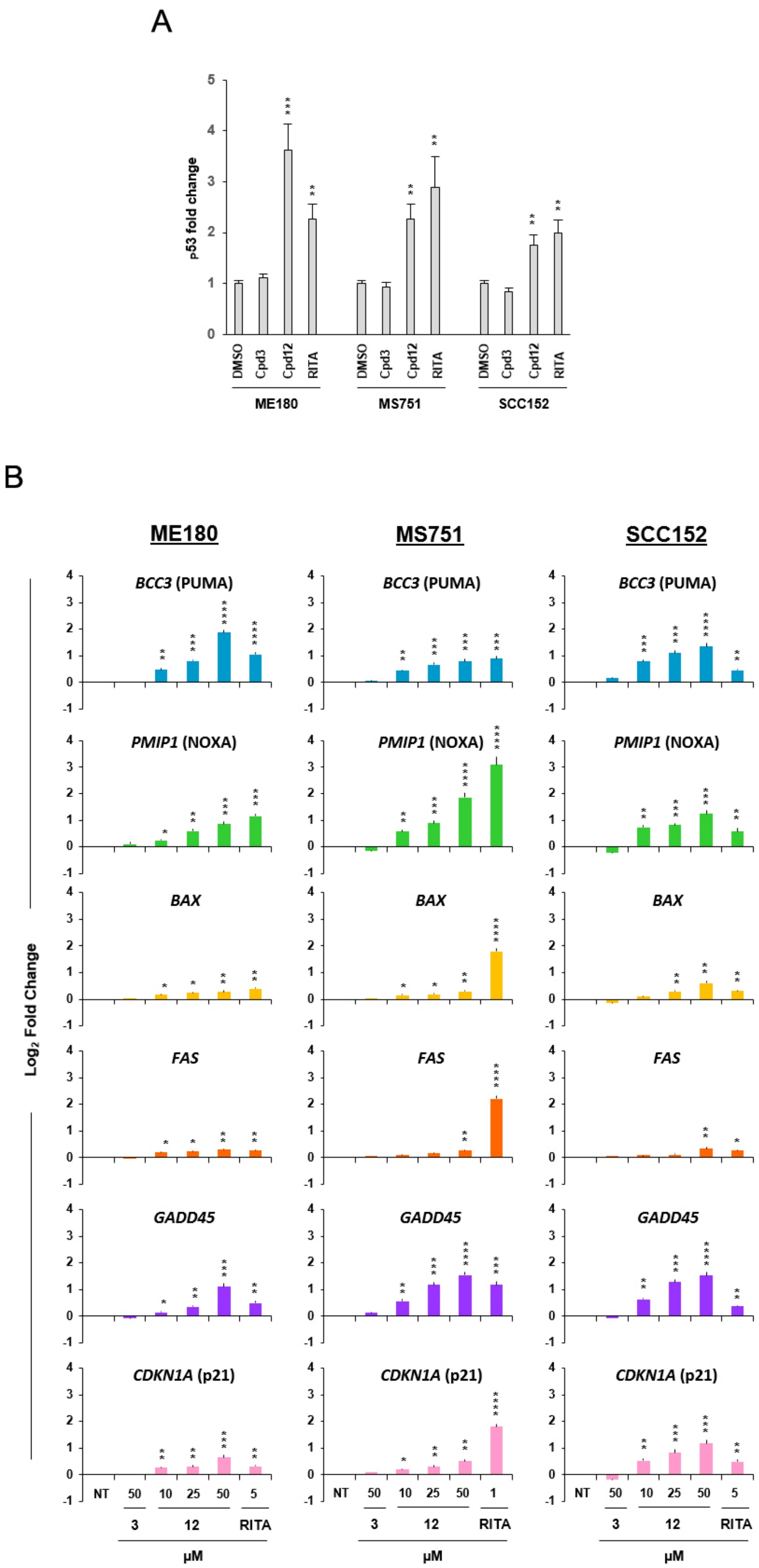
A
p53 fold change
DMSO
Cpd3
Cpd12
RITA
ME180
MS751
SCC152
B
ME180
MS751
SCC152
BCC3 (PUMA)
PMIP1 (NOXA)
BAX
FAS
GADD45
CDKN1A (p21)
Log2 Fold Change
NT
3
12
RITA
µM

Figure S3. Cpd12 rescues p53 levels and transcriptional activity in both cervical and head-and-neck cancer cells.

(A) Bar graph showing the quantification of p53 protein bands, after normalization to the relative β-actins, of the Western blot analysis shown in Figure 1C. Data represent the mean ± SD of three independent experiments. Quantifications were performed with ImageJ and data were analyzed by a one-way ANOVA followed by Tukey's multiple comparison test. **, $p < 0.01$; ***, $p < 0.001$; Cpd12/RITA *versus* DMSO.

(B) Bar graphs showing the non-normalized $\log_2$ fold-change values of p53-target gene expression in ME180, MS751, and SCC152 cells upon compound treatment presented as heatmaps in Figure 1D. Data represent the mean ± SD of three independent experiments and were analyzed by a two-way ANOVA followed by Tukey's multiple comparison test. *, $p < 0.05$; **, $p < 0.01$; ***, $p < 0.001$; ****, $p < 0.0001$ Cpd12/RITA *versus* DMSO

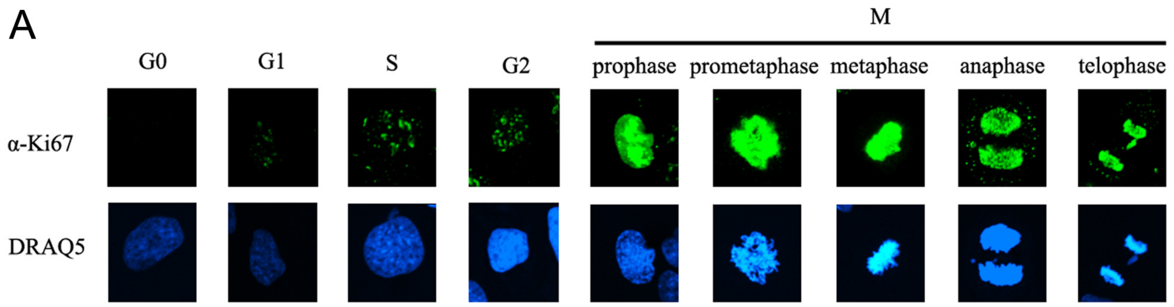

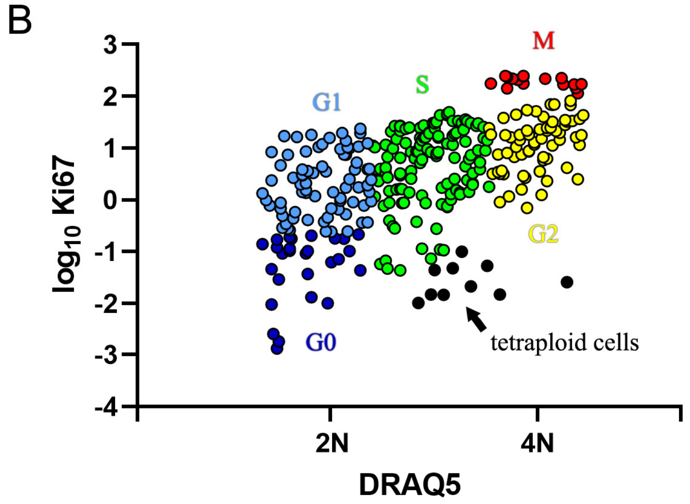

Figure S4. The cell-cycle arrest induced by Cpd12 occurs in the G1 phase.

(A) Representative confocal images of different untreated HeLa cells in different phases of the cell cycle. The images show the relative increase of Ki67 and nuclear fluorescence intensities according to the cell-cycle phase in which cells were blocked upon fixation. Images show the maximum intensity projections of Z-stacks acquired with a 600 × magnification.

(B) Representative semi-log plot of untreated HeLa cells and the relative distributions of cells in the different phases of the cell cycle, determined as a function of Ki67 and nuclear fluorescence intensities as shown in **(A)**. Ki67 fluorescence intensities quantified from maximum intensity projections are expressed on a logarithmic scale, while DRAQ5 fluorescence intensities are expressed on a linear scale. Clustering of the different cell populations was defined as reported by Miller and colleagues [17].

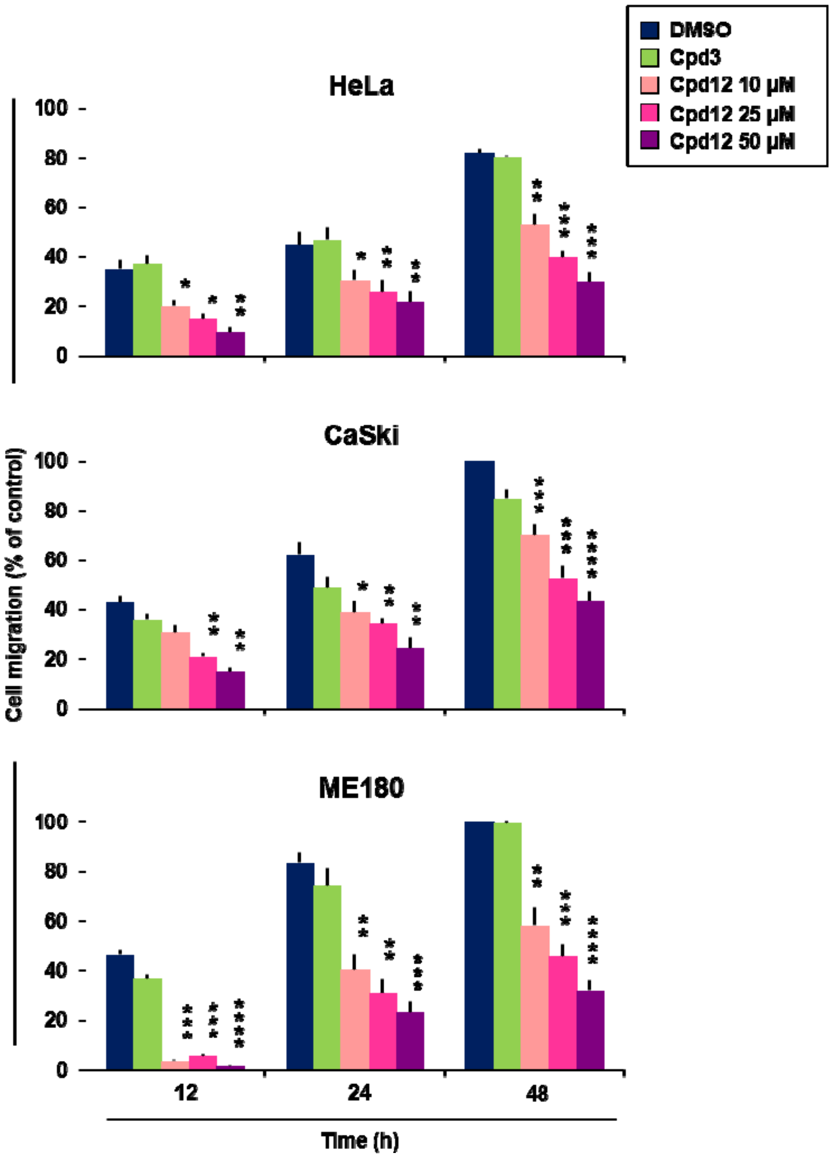

Figure S5. The Cpd12-induced rescue of p53 affects the migration capacity of HPV-transformed cells.

Migration rates of HeLa, CaSki, and ME180 cells shown in Figure 4A expressed as the distance between the edges of the wound (defined by the lines in Figure 4A) following compound treatments. Values in the bar graph indicate the surface areas occupied by the migrating cells after 12, 24, and 48 hours, where 100% indicates complete wound closure, and represent the mean ± SD of three different experiments. Data were analyzed by a two-way ANOVA followed by Tukey's multiple comparison test. *, $p < 0.05$; **, $p < 0.01$; ***, $p < 0.001$; ****, $p < 0.0001$ Cpd12 *versus* DMSO.

Supplementary Information

Original uncropped blots displayed in Figure 1C. βactin and p53 were blotted onto the same membrane and were visualized separately after cutting the membrane.

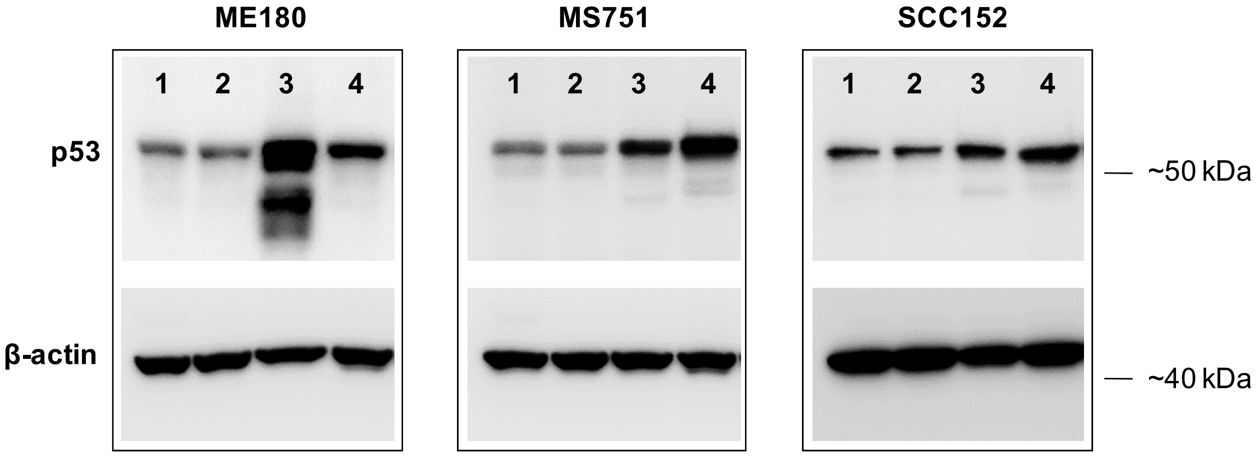

1: DMSO; 2: Cpd3; 3: Cpd12; 4: RITA.
